# PKMYT1 in Cancer: Beyond Cell Cycle Checkpoints to Context‐Dependent Therapeutic Vulnerability

**DOI:** 10.1002/gcc.70151

**Published:** 2026-07-01

**Authors:** Lingxi Li, Binfan He, Mengmeng Hao, Ye Liu, Shunxin Song, Rongzhang He

**Affiliations:** ^1^ Department of General Surgery Translational Medicine Institute, the Affiliated Chenzhou Hospital, Hengyang Medical School, University of South China Chenzhou China

**Keywords:** CCNE1 amplification, Lunresertib (RP‐6306), PKMYT1, Precision oncology, Replication stress, Rynthetic lethality

## Abstract

PKMYT1 has emerged as a promising therapeutic target distinguished by its tumor‐selective expression and essential role in replication stress management. Unlike WEE1, PKMYT1 is dispensable in normal cell cycles but critical for cancer cells coping with DNA damage, establishing a broad therapeutic window. This vulnerability is exemplified by synthetic lethality in CCNE1‐amplified and TP53‐deficient contexts, where PKMYT1 inhibition triggers catastrophic mitotic entry. Beyond canonical cell cycle regulation, PKMYT1 functions as a multifaceted oncoprotein modulating signaling networks, metabolic reprogramming, and immune evasion via cGAS‐STING activation. With selective inhibitors like lunresertib (RP‐6306) now in Phase I/II trials, often combined with ATR inhibitors or chemotherapy, the field stands at a translational inflection point. However, context‐dependent roles (e.g., tumor‐suppressive functions in LUAD) and undefined resistance mechanisms pose challenges. This review critically evaluates PKMYT1's mechanistic underpinnings, clinical landscape, and biomarker strategies. We advocate for precision targeting based on genetic signatures (CCNE1, TP53, ER) to optimize therapeutic efficacy and overcome resistance in replication stress‐high malignancies.

**Abbreviations**
BC: Breast cancer
CCNB1: Cyclin B1
CDKs: Cyclin‐dependent kinases
CRC: Colorectal cancer
DDR: DNA damage response
EMT: Epithelial‐mesenchymal transition
GC: Gastric cancer
HCC: Hepatocellular carcinoma
LUAD: Lung adenocarcinoma
NSCLC: Non‐small cell lung cancer
OC: Ovarian cancer
PKMYT1: Protein kinase, membrane‐associated tyrosine/threonine 1
TNBC: Triple‐negative breast cancer
WEE1: WEE1 G2 checkpoint kinase

## 1 Introduction

Replication stress (RS) driven genomic instability is a core feature of tumorigenesis [1]. When replication fork stalling leads to single‐stranded DNA (ssDNA) exposure, cells activate checkpoint kinases, notably WEE1 and PKMYT1, to enforce a G2/M arrest and prevent premature mitotic entry, thereby avoiding mitotic catastrophe [2, 3]. However, cancer cells often harbor partial defects in the DNA damage response (DDR). The DDR arrests the cell cycle to allow time for DNA repair and, if damage is irreparable, triggers elimination pathways, thereby preserving genomic integrity and preventing carcinogenesis [4, 5]. Mechanistically, DNA damage sensors such as ATM and ATR initiate signaling cascades that stabilize p53 and E2F1, determining cell fate between repair‐mediated arrest and caspase‐dependent apoptosis [6]. However, p53 signaling is impaired in approximately 50% of human malignancies, enabling tumor cells to bypass G1 arrest and evade apoptotic elimination despite accumulating DNA damage [7]. Although this defect allows tumor cells to survive under chronic RS, it traps them in a “fragile balance”: once compensatory repair pathways are disrupted, genomic instability accelerates sharply, leading to cell death. While conventional radiotherapy and chemotherapy induce DNA damage, they are limited by a lack of selectivity; therefore, synthetic lethality strategies targeting tumor‐specific genetic vulnerabilities have become a frontier direction in precision oncology [8].

PKMYT1 has emerged as a critical node within this synthetic lethality network. Although PKMYT1 belongs to the WEE kinase family like WEE1, and both inhibit CDK1 activity to prevent Cyclin B binding and mitotic entry, their subcellular localization and functional redundancy differ significantly [9]. WEE1 is primarily nuclear, whereas PKMYT1 is membrane‐tethered to the Golgi and endoplasmic reticulum, sequestering CDK1‐Cyclin B complexes in the cytoplasm [10, 11, 12, 13, 14]. Mechanistically, PKMYT1 uniquely phosphorylates CDK1 at both Thr14 and Tyr15 residues and contributes to this protective mechanism by enforcing cell cycle arrest in response to DNA damage and facilitating checkpoint maintenance through regulation of CDK activity [15, 16, 17]. Recent insights reveal that the ATR‐CHK1 axis exerts phase‐specific regulatory effects on these kinases. In interphase, ATR‐CHK1 signaling enforces G2/M arrest primarily through WEE1 activation. Conversely, in mitosis, ATR is constitutively active due to centromeric R‐loops, and CHK1 directly phosphorylates PKMYT1 to sustain CDK1 activity for proper chromosome segregation. This phase‐specific switch is governed by nuclear envelope breakdown, which removes the spatial barrier separating CHK1 and PKMYT1 during interphase [18]. While WEE1 predominantly regulates CDK2 to prevent RS in normal cycles, PKMYT1 becomes critical under specific oncogenic conditions. For instance, glioblastoma stem cells lose the functional redundancy between PKMYT1 and WEE1, exhibiting specific dependence on PKMYT1 inhibition [19, 20]. This tumor‐specific dependency, coupled with its dynamic regulation by ATR‐CHK1, provides a broad therapeutic window.

Beyond cell cycle regulation, PKMYT1 has been found to exhibit characteristics of a multifunctional oncoprotein involved in metabolic reprogramming, post‐transcriptional regulation, and drug resistance. In contrast to the ubiquitous expression of WEE1 in somatic cells, PKMYT1 is minimally expressed in normal tissues yet markedly upregulated in diverse malignancies [21], highlighting its promise as a tumor‐selective therapeutic target with a favorable therapeutic window. Given its druggable kinase domain and key status in synthetic lethal interactions, PKMYT1 represents a potential target for next‐generation precision therapy. This review aims to systematically elaborate on the molecular mechanisms, prognostic significance, and clinical translation potential of PKMYT1, with a particular focus on its latest research progress as a synthetic lethality target, providing a theoretical basis for developing novel therapies for DDR‐deficient tumors.

## 2 PKMYT1 as a Regulator of Replication Stress Tolerance: Normal Versus Malignant Cells

### 2.1 PKMYT1 Mechanisms in Normal Cell Cycle Regulation

In normal cells, PKMYT1 acts as a safeguard kinase maintaining genomic integrity. Ruiz et al. demonstrated that PKMYT1 keeps CDK1‐cyclin B complexes inactive via dual phosphorylation at Thr14 and Tyr15, distinct from WEE1, which targets Tyr15 exclusively. Notably, PKMYT1 inhibition alone is sufficient to trigger entry into meiosis I [22, 23]. However, in unperturbed somatic cells, PKMYT1 is largely dispensable. Chow et al. reported that PKMYT1 depletion does not alter baseline cell cycle timing in normal conditions but significantly sensitizes cells to DNA damaging agents [20]. This supports a model where PKMYT1 is redundant in normal tissue homeostasis yet becomes essential under stress. In contrast, cancer cells often harbor G1 checkpoint defects and rely heavily on the G2/M checkpoint for survival. Functional redundancy between PKMYT1 and WEE1 exists in normal neural stem cells but is lost in glioblastoma, creating a tumor‐specific vulnerability [19]. Although PKMYT1 is dispensable for routine cell cycle progression, it serves as a rate‐limiting factor during recovery from the G2/M checkpoint following DNA damage [19, 20]. These findings underscore the therapeutic potential of PKMYT1 inhibition. Its non‐essential role in unperturbed cell cycles, coupled with its critical function in checkpoint recovery after DNA damage, indicates that targeting PKMYT1 may selectively compromise cancer cells‐particularly those with elevated replication stress or DNA damage while sparing normal tissues. This context‐dependent vulnerability positions PKMYT1 as a promising target for precision cancer therapy.

### 2.2 PKMYT1 Mechanisms in Tumor Cell Cycle Regulation

**FIGURE 1:**
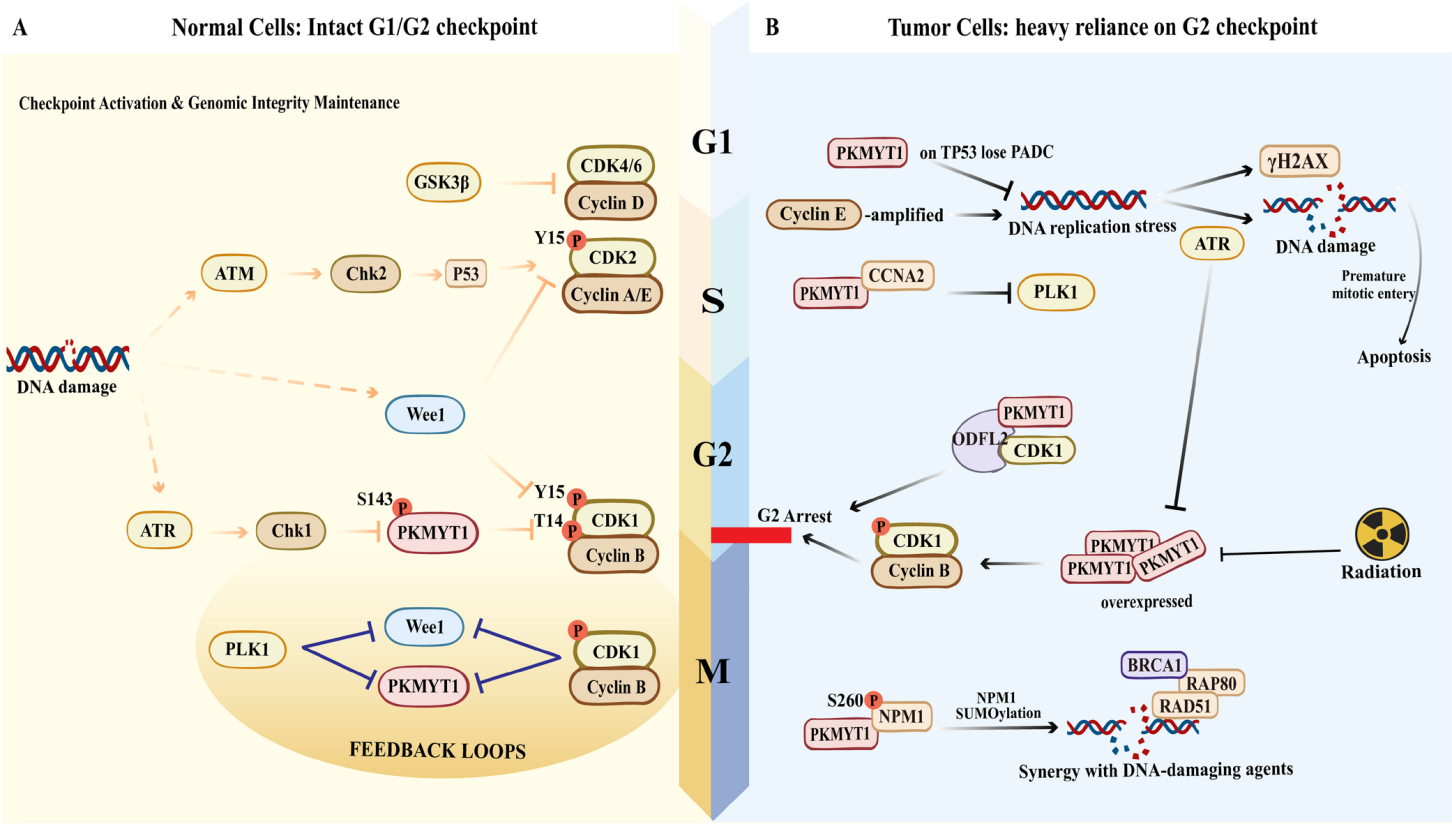
PKMYT1‐mediated cell cycle checkpoint regulation in normal versus tumor cells. Panel A: Normal cells maintain intact G1/G2 checkpoints. DNA damage activates ATR‐Chk1‐PKMYT1 signaling, leading to CDK1‐Thr14/Tyr15 phosphorylation and G2 arrest. PLK1‐WEE1‐PKMYT1 feedback loops regulate CDK1‐Cyclin B activity, ensuring genomic integrity. Panel B: Tumor cells with G1 defects depend on PKMYT1 for G2 checkpoint recovery. Oncogenic drivers (CCNE1 amplification, TP53 loss) induce replication stress and PKMYT1 overexpression. PKMYT1 stabilizes CCNA2, interacts with PLK1/ODFL2, and impairs DDR via NPM1‐Ser260 phosphorylation (reducing BRCA1/RAD51 recruitment). Inhibition forces premature mitotic entry, causing mitotic catastrophe and apoptosis. Radiation and DNA‐damaging agents enhance this effect. PKMYT1 is nonessential in normal cells but critical in cancer cells with replication stress, establishing a therapeutic window.

Cancer cells, often harboring G1 checkpoint defects, rely heavily on the G2/M checkpoint to prevent premature mitotic entry. Consequently, pharmacological abrogation of the G2 checkpoint has emerged as a promising strategy to selectively target cancer cells [24, 25]. PKMYT1 overexpression disrupts the nuclear‐cytoplasmic shuttling of the CDK1‐cyclin B1 complex, leading to G2‐phase arrest [26, 27]. Accumulating evidence indicates that PKMYT1 is overexpressed in a wide range of cancers, correlating with advanced TNM stage and poor prognosis. Critically, PKMYT1 dependency demonstrates potent efficacy in specific genetic contexts. In lung adenocarcinoma (LUAD), PKMYT1 depletion abrogates radiation‐induced G2/M arrest, reducing clonogenic survival [28]. In epithelial ovarian cancer (OC), the centrosomal protein ODF2L bridges PKMYT1 and CDK1 via its N‐terminal and C‐terminal domains, respectively, forming a ternary complex that enables PKMYT1 to phosphorylate and inactivate CDK1, thereby enforcing G2/M arrest [29]. Importantly, when WEE1 is pharmacologically inhibited, PKMYT1‐mediated CDK1 inactivation becomes critical for checkpoint maintenance, revealing a compensatory role for PKMYT1 in G2/M control. Notably, CCNE1‐amplified OC‐a subtype characterized by replication stress‐exhibits heightened sensitivity to dual targeting of PKMYT1 and ATR pathways. Synergistic enhancement of cytotoxicity is observed in CCNE1‐amplified OC and endometrial cancer, with minimal effect on non‐amplified counterparts [30]. Moreover, PKMYT1 represents a synthetic lethal target in the context of cyclin E1 (CCNE1) overexpression, a common feature in a subset of CRC. Co‐occurrence of CCNE1 amplification and PKMYT1 loss leads to accumulation of DNA double‐strand breaks, as indicated by elevated γH2AX levels, persistent activation of the DDR and subsequent apoptosis [31]. Mechanistically, PKMYT1 directly binds to cyclin A2 (CCNA2)‐a key regulator of S/G2 transition‐and stabilizes its expression through protein‐protein interaction; knockdown of PKMYT1 reduces CCNA2 levels, thereby suppressing oncogenic phenotypes [32]. In pancreatic ductal adenocarcinoma (PDAC), PKMYT1 is markedly overexpressed and functions as an oncogene by interacting with CDK1 and PLK1 to drive tumorigenesis. Inhibition induces premature mitotic entry, leading to unrepaired DNA damage and pan‐nuclear γH2AX accumulation‐hallmarks of mitotic catastrophe. Notably, sensitivity to PKMYT1‐targeted therapy is significantly enhanced by TP53 loss and further modulated by PRKDC (DNA‐PKcs) activity, highlighting these molecular features as potential biomarkers for patient stratification in PDAC [33]. In prostate cancer, PKMYT1 promotes growth by upregulating key cell cycle regulators CCNB1 and CCNE1 through transcriptional activation, showing responsiveness to kinase inhibition [34]. Additionally, PKMYT1 induces phosphorylation of nucleophosmin 1 (NPM1) at the S260 site, which competitively impairs NPM1 SUMOylation. This modification interferes with the recruitment of essential DNA damage response factors, including BRCA1, RAP80, and RAD51, to DNA damage sites, ultimately affecting homologous recombination‐mediated DSB repair. Synergy with DNA‐damaging agents is observed in osteosarcoma (OS) cells [35]. Collectively, these data validate PKMYT1 as a precision medicine target whose inhibition selectively kills cancer cells relying on checkpoint recovery (Figure 1).

## 3 Pleiotropic Oncogenic Functions Beyond Cell Cycle Control

### 3.1 Integrator of Oncogenic Signaling Networks

Beyond cell cycle regulation, PKMYT1 functions as a signaling hub driving malignancy across multiple cancer types. In prostate cancer, E2F1 regulates PKMYT1 expression, and PKMYT1 inhibits the PPAR signaling pathway, reinforcing tumor progression [36]. In gastric cancer (GC), high PKMYT1 expression independently predicts poor prognosis. PKMYT1 enhances proliferation and apoptosis resistance by activating the MAPK pathway [37]. The E2F7‐PKMYT1 axis promotes GC proliferation and attenuates Adriamycin sensitivity via MAPK activation [38]. Mechanistically, E2F7 transcriptionally upregulates PKMYT1, and silencing E2F7 restores drug sensitivity, suggesting a potential combinatorial targeting strategy.

In non‐small cell lung cancer (NSCLC) and triple‐negative breast cancer (TNBC), PKMYT1 sustains malignancy via Notch signaling activation; its inhibition downregulates NOTCH1/HES1 and reverses epithelial‐mesenchymal transition (EMT) [39, 40]. In hepatocellular carcinoma (HCC) and esophageal squamous cell carcinoma (ESCC), PKMYT1 silencing suppresses growth via downregulation of MAPK/ERK and PI3K/AKT/mTOR axes [41, 42]. However, context‐dependency defines PKMYT1 biology. In a LUAD subset, PKMYT1 acts as a tumor suppressor by inhibiting AKT1 phosphorylation at Thr308 [43]. This dichotomy underscores the need for biomarker‐driven patient stratification.

### 3.2 Regulator of Metabolic Reprogramming and Immune Evasion

Beyond cell cycle control, PKMYT1 contributes to metabolic reprogramming in GC. PKMYT1 acts downstream of the transcription factor TEAD4. PKMYT1 overexpression increases glycolytic metabolites (pyruvate, lactate) and enzymes (HK‐2, PKM2), effects rescued by TEAD4 inhibition [44]. Crucially, PKMYT1 inhibition activates the cGAS‐STING pathway, promoting interferon signaling and CD8^+^ T cell infiltration. The inhibitor RP‐6306 enhances immune checkpoint blockade efficacy, positioning PKMYT1 as a dual regulator of tumor‐intrinsic growth and immune evasion in CRPC [45].

### 3.3 Driver of Metastasis and EMT

PKMYT1 is overexpressed in breast cancer (BC) and drives aggressive tumor phenotypes [46, 47]. In breast cancer, elevated PKMYT1 correlates with aggressive subtypes (ER‐negative, TNBC) and co‐upregulation with PLK1 validates both as prognostic markers [48].

In hepatocellular carcinoma (HCC), PKMYT1 drives progression by promoting cell growth, migration, and EMT. Mechanistically, PKMYT1 directly binds to and inactivates GSK3β, disrupting its interaction with β‐catenin. This stabilizes β‐catenin, leading to hyperactivation of the β‐catenin/TCF transcriptional program [49]. In colorectal cancer (CRC), PKMYT1 depletion impairs proliferation, migration, and invasion [23]. In ovarian cancer (OC), PKMYT1 upregulation correlates with distant metastasis and poor prognosis. The mitochondrial deacetylase SIRT3 exhibits a negative correlation with PKMYT1 expression, and silencing SIRT3 abrogates the tumor‐suppressive effects of PKMYT1 depletion [50]. In oral squamous cell carcinoma (OSCC) and clear cell renal cell carcinoma (ccRCC), PKMYT1 knockdown suppresses proliferation, migration, and invasion and reverses the EMT phenotype [32, 51, 52]. PKMYT1 induces EMT through the FoxM1/Snail axis, repressing E‐cadherin and activating vimentin. Inhibition of this pathway restores E‐cadherin levels and reverses metastatic phenotypes, validating it as a therapeutic target [53].

## 4 Mechanisms of PKMYT1 Dysregulation in Cancer

### 4.1 Genetic and Epigenetic Alterations

PKMYT1 expression is tightly regulated through epigenetic mechanisms. Zhang et al. demonstrated that upregulation of the histone demethylase KDM2B represses let‐7b‐5p, leading to increased EZH2 expression, which transcriptionally activates PKMYT1 [54]. Ectopic co‐expression of EZH2 and PKMYT1 reversed the tumor‐suppressive effects of KDM2B knockdown and accelerated NSCLC cell proliferation, migration, and invasion. Notably, EZH1 exerts an opposing effect: in breast cancer, EZH1 transcriptionally represses PKMYT1, and its downregulation contributes to TNBC progression by derepressing PKMYT1 expression‐a mechanism also observed in lung cancer [55]. This EZH1/EZH2 dichotomy highlights context‐specific epigenetic control.

### 4.2 Post‐Transcriptional Control

Post‐transcriptional mechanisms further fine‐tune PKMYT1 expression. In gastric cancer, PKMYT1 promotes invasion in an m^6^A‐dependent manner [56]. ALKBH5, an m^6^A demethylase, removes m^6^A modifications from PKMYT1 mRNA, enabling recognition by the reader protein IGF2BP3, which stabilizes the transcript and enhances metastatic potential. In NSCLC, the lncRNA PKMYT1AR is significantly upregulated and correlates with adverse clinical outcomes. PKMYT1AR functions as a molecular sponge for miR‐485‐5p, attenuating repression of PKMYT1. Consistently, miR‐485‐5p is downregulated in NSCLC cell lines and patient serum, and its forced expression inhibits tumor proliferation and migration. Targeting PKMYT1AR with antisense oligonucleotides (ASOs) markedly suppresses tumor growth in vivo. In osteosarcoma, miR‐601 directly targets PKMYT1; their inverse correlation predicts prognosis [57, 58]. High PKMYT1 expression predicts poor prognosis in OS patients and exacerbates malignancy, potentially via NF‐κB pathway regulation [59]. Additionally, miR‐497‐5p co‐inhibits PKMYT1 and WEE1, suggesting a synthetic lethal interaction in dedifferentiated pleomorphic sarcoma [60].

### 4.3 Protein Stability and Interaction

At the protein level, MCRS1 physically interacts with PKMYT1, reducing its stability and modulating cell cycle progression in GC [61]. MCRS1 overexpression reduces PKMYT1 protein levels, upregulates CCND1, and downregulates CCNB1, along with decreased CDK1 phosphorylation at Thr14 and Tyr15. Treatment with MK‐1775, a selective PKMYT1 inhibitor, phenocopies the effects of MCRS1 overexpression. Conversely, USP49 limits PKMYT1 ubiquitination and degradation [62]. Overexpression of PKMYT1 restores malignant phenotypes in USP49‐deficient TNBC cells, uncovering a novel USP49‐PKMYT1 regulatory axis.

**FIGURE 2:**
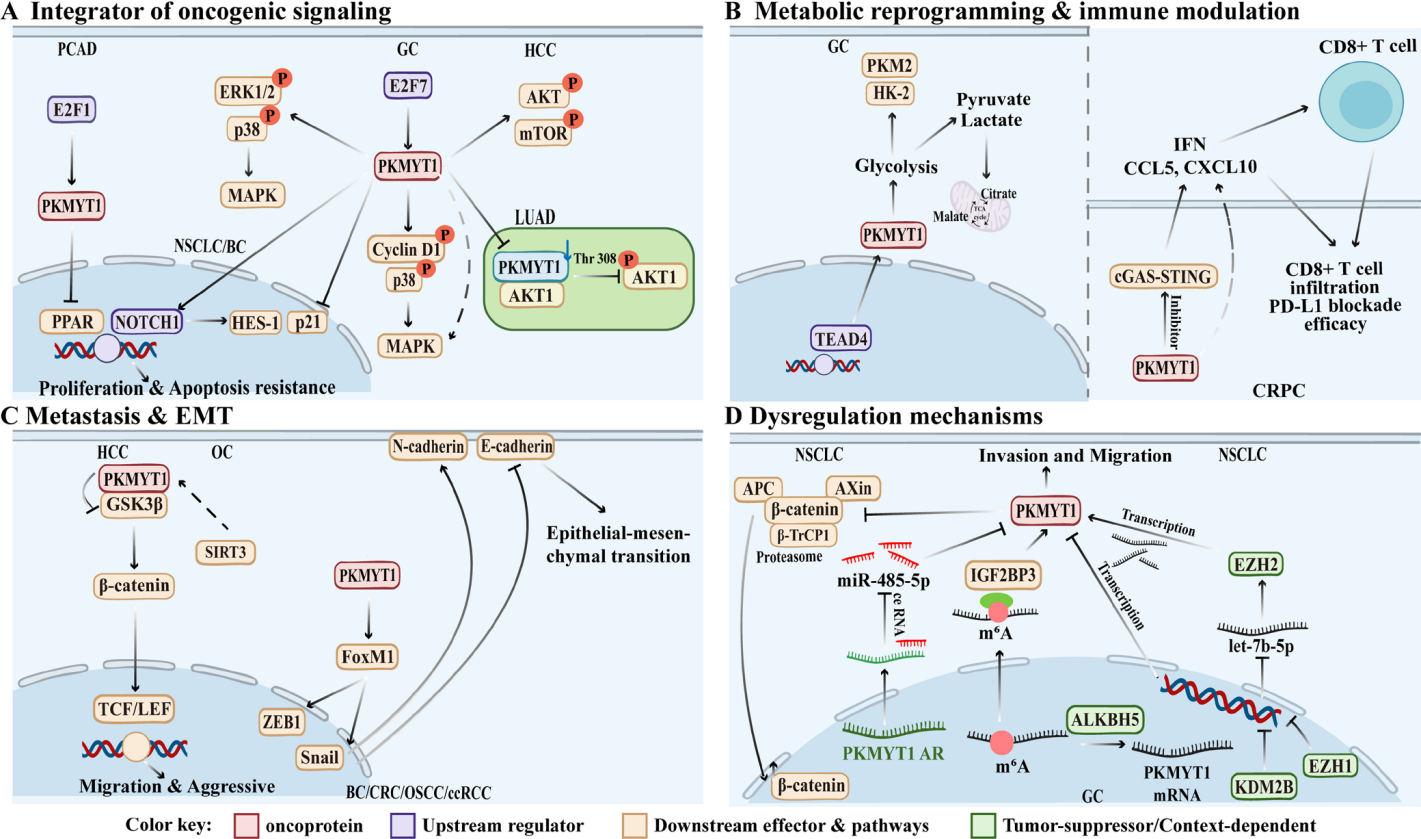
PKMYT1 functions as a multifunctional oncoprotein beyond cell cycle control. Panel A: PKMYT1 orchestrates oncogenic signaling across cancers. It activates MAPK/ERK (GC), AKT/mTOR (HCC), and NOTCH1 (NSCLC/BC), promoting proliferation and apoptosis resistance. Context‐dependently, PKMYT1 suppresses AKT1‐Thr308 in LUAD, highlighting tumor‐type‐specific functions. Panel B: PKMYT1 reprograms metabolism via TEAD4‐driven glycolysis (HK‐2/PKM2 upregulation). Immunologically, PKMYT1 inhibition activates cGAS‐STING, potentiating interferon signaling and CD8^+^ T cell recruitment, sensitizing tumors to immune checkpoint blockade. Panel C: PKMYT1 drives metastasis through β‐catenin stabilization (GSK3β inactivation, HCC) and FoxM1‐Snail‐mediated EMT (BC/CRC/ccRCC), repressing E‐cadherin and inducing mesenchymal markers. Panel D: Multilevel regulation governs PKMYT1 expression: Epigenetic (KDM2B/EZH2), post‐transcriptional (m^6^A/ALKBH5, lncRNA/miRNA), and post‐translational (MCRS1/USP49‐mediated stability). PKMYT1's pleiotropic functions support combination strategies targeting signaling, metabolism, and immunity.

In summary, PKMYT1 is governed by a multilayered regulatory network encompassing epigenetic, post‐transcriptional, and post‐translational mechanisms. Understanding these regulatory layers offers opportunities for biomarker development and combinatorial therapeutic strategies (Table 1, Figure 2).

**TABLE 1:** Expression of PKMYT1 and its functional characterizations in human cancers.

Mechanism category	Key finding	Cancer types	Role	Clinical correlation	References
Replication stress tolerance	CCNE1 amp → PKMYT1 dependency; synthetic lethality with WEE1 loss	OC, CRC, EMCA	Oncogene	Poor OS, HR = 2.1	[30, 63]
G2/M checkpoint control	Rate‐limiting factor for checkpoint recovery; Dispensable in normal cells	Pan‐cancer	Oncogene	Radioresistance	[19, 20]
CDK1 regulation	Dual phosphorylation (Thr14/Tyr15); loss of redundancy with WEE1	GBM, PDAC	Oncogene	TP53 loss enhances sensitivity	[12, 13, 14]
MAPK/ERK Signaling	Activates MAPK pathway; E2F7‐PKMYT1 axis	GC, NSCLC, HCC, ESCC	Oncogene	Advanced stage; Poor prognosis	[37, 38]
PI3K/AKT/mTOR pathway	Activates AKT/mTOR signaling; in LUAD: binds AKT1, inhibits p‐Thr308	HCC, ESCC, ccRCC, LUAD	Oncogene/Tumor suppressor	Context‐dependent: Tumor suppressor in LUAD	[41, 42, 43]
Notch signaling	Activates NOTCH1/HES1; Regulates p21	NSCLC, TNBC	Oncogene	EMT promotion; Drug resistance	[39, 40]
Wnt/β‐catenin pathway	Binds/inactivates GSK3β → β‐catenin stabilization	HCC, NSCLC	Oncogene	Advanced TNM stage; Poor OS	[49]
Metabolic reprogramming	TEAD4‐PKMYT1 axis → ↑Glycolysis (HK‐2, PKM2); ↑Pyruvate, lactate	GC	Oncogene	Warburg effect; Enhanced metastasis	[44]
Immune modulation	Inhibition → cGAS‐STING activation → ↑IFN signaling; ↑CCL5, CXCL10	CRPC	Oncogene	CD8^+^ T cell infiltration; ICB sensitization	[45]
EMT/metastasis	FoxM1/Snail axis → ↓E‐cadherin; ↑N‐cadherin, Vimentin, ZEB1	ccRCC, BC, CRC, OSCC, ESCC	Oncogene	Metastasis; EMT markers; Poor survival	[40, 49, 53]
DR and NPM1 regulation	Phosphorylates NPM1 at S260 → Impairs SUMOylation; ↓BRCA1, RAP80, RAD51 recruitment	OS	Oncogene	Cisplatin sensitization	[35]
CCNA2 stabilization	Directly binds/stabilizes Cyclin A2	OC, CRC	Oncogene	CCNE1 amplification biomarker	[32]
Epigenetic regulation	KDM2B/let‐7b/EZH2 axis → ↑PKMYT1; EZH1 represses PKMYT1 in TNBC	NSCLC, TNBC	Oncogene	EZH2/EZH1 as prognostic markers	[54, 55]
m^6^A modification	ALKBH5 → m^6^A demethylation → IGF2BP3 stabilizes PKMYT1 mRNA	GC	Oncogene	Metastasis; IGF2BP3 correlation	[56]
lncRNA regulation	PKMYT1AR sponges miR‐485‐5p → ↑PKMYT1; ↑β‐catenin via ↓β‐TrCP1	NSCLC	Oncogene	CSC maintenance; Poor prognosis	[57]
miRNA regulation	miR‐601, miR‐497‐5p target PKMYT1; Synthetic lethality with WEE1 inhibition	OS, DPM	Oncogene	Inverse correlation with miRNAs	[58, 59, 60]
Protein stability	MCRS1 ↓PKMYT1 protein; USP49 prevents ubiquitination and degradation	GC, TNBC	Oncogene	USP49 as protective factor	[61, 62]
Therapeutic targeting	Lunresertib (RP‐6306) + Camonsertib synergy; fostamatinib inhibition	OC, EMCA, PDAC, Prostate	Drug target	Combination therapy efficacy; biomarker‐guided	[30, 33, 34, 63, 64, 65, 66, 67, 68]

## 5 Clinical Translation and Therapeutic Strategies

### 5.1 Synthetic Lethality Mechanisms: Rationale for PKMYT1 Targeting

Oncology drug discovery based on synthetic lethal interactions holds great promise, yet few candidates have been developed ab initio using this approach. A genome‐scale genetic interaction screen in a CCNE1‐amplified cellular model identified PKMYT1 inhibition as a selective vulnerability, leading to the discovery of RP‐6306, a selective PKMYT1 kinase inhibitor. Mechanistically, the observed synthetic lethality between PKMYT1 and CCNE1 amplification may result from a two‐stage activation model: CCNE1‐driven DNA replication stress and MMB‐FOXM1 transcription increase cyclin B‐CDK1 levels and activity in S phase, rendering cells highly vulnerable to loss of PKMYT1‐driven inhibitory CDK1 Thr14 phosphorylation [63]. The resulting CDK1 activation causes unscheduled mitotic entry and mitotic‐interphase oscillations associated with catastrophic genome instability. Notably, PKMYT1‐but not WEE1‐shows synthetic lethality with CCNE1 amplification, likely owing to PKMYT1's selectivity for CDK1 (vs. WEE1's activity toward both CDK1 and CDK2) [11, 60, 69].

### 5.2 Small‐Molecule PKMYT1 Inhibitors: From Bench to Bedside

RP‐6306 (lunresertib) is an orally bioavailable, selective PKMYT1 inhibitor that has entered first‐in‐human clinical studies as monotherapy (NCT04855656) and in combination with gemcitabine (NCT05147272) or FOLFIRI (NCT05147350) [63, 64, 65]. Emerging advances in targeted cancer therapy include XH‐30, which demonstrates antitumor efficacy in P53‐mutated TNBC models and synergizes with the PARP inhibitor Olaparib [66]; Compound 7, a pyrrolopyrimidinone derivative with enhanced metabolic stability [67]; A30, a pyrimidinyl amide optimized via molecular dynamics‐guided design [65]. Additionally, the PROTAC D16‐M1P2 achieves dual PKMYT1 degradation and inhibition, exhibiting robust antitumor responses in xenograft models [68] and the mechanistic elucidation of RX‐3117 resistance, which identifies NT5C3‐mediated deactivation‐not impaired activation enzymes‐as the primary driver of nucleoside analogue resistance in NSCLC [70]. Structural studies reveal that PKMYT1's narrower ATP‐binding pocket may confer relative resistance to WEE1‐targeted inhibitors, providing a rationale for structure‐guided design of selective PKMYT1 inhibitors [71, 72].

### 5.3 Rational Combination Strategies

PKMYT1 inhibition demonstrates synergy with multiple therapeutic modalities. Co‐administration of RP‐6306 with the ATR inhibitor RP‐3500 (camonsertib) synergistically enhances cytotoxicity in CCNE1‐amplified cells by promoting premature mitosis and DNA damage [30, 73]. Combination with DNA‐damaging agents (gemcitabine, cisplatin, hydroxyurea) enhances replication stress and sensitizes tumors to PKMYT1 inhibition. PKMYT1 inhibition also activates the cGAS‐STING pathway, potentiating interferon signaling and CD8^+^ T cell infiltration; combining RP‐6306 with PD‐L1 blockade significantly increases antitumor efficacy in castration‐resistant prostate cancer [45]. In TP53‐mutated TNBC, PKMYT1 inhibition facilitates G2/M phase release, overcoming PARP inhibitor‐induced G2 arrest and yielding synergistic antitumor effects with Olaparib [66].

### 5.4 Biomarkers for Patient Stratification

**FIGURE 3:**
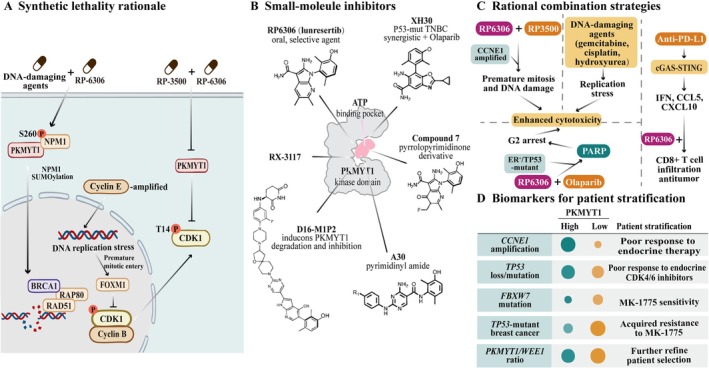
PKMYT1‐targeted therapy: from synthetic lethality to precision oncology. Panel A: Synthetic lethality arises in CCNE1‐amplified tumors where replication stress creates PKMYT1 dependency. Inhibition triggers premature mitosis, DNA damage, and impaired DDR. ATR inhibition synergizes by amplifying replication stress. Panel B: Multiple PKMYT1 inhibitors are in development. RP‐6306 leads clinical trials. XH‐30 targets P53‐mutant TNBC with PARP synergy. Compound 7 and A30 offer novel scaffolds with improved stability/selectivity. D16‐M1P2 achieves dual degradation/inhibition via PROTAC technology. Panel C: Rational combinations maximize efficacy: ATR inhibitors (synthetic lethality), chemotherapy (replication stress amplification), immunotherapy (cGAS‐STING activation enhancing CD8^+^ T cell infiltration), and PARP inhibitors (overcoming G2 arrest in TP53‐mutant cancers). Panel D: Biomarker‐driven stratification uses CCNE1 amplification, TP53/FBXW7 mutations, ER status, and PKMYT1/WEE1 ratios to identify responsive populations. PKMYT1 inhibition represents a biomarker‐driven precision therapy with multiple combination opportunities.

Several biomarkers may identify patients most likely to benefit from PKMYT1 inhibition. Genetic alterations include CCNE1 amplification, TP53 loss, FBXW7 mutation, and ER^+^/TP53‐mutant status in breast cancer [63]. Elevated PKMYT1 mRNA levels correlate with poor response to endocrine therapy and CDK4/6 inhibitors in ER^+^ breast cancer [74]. Low PKMYT1 expression may predict sensitivity to the WEE1 inhibitor MK‐1775, whereas PKMYT1 upregulation is associated with acquired resistance to MK‐1775 [66, 69]. The PKMYT1/WEE1 expression ratio may further refine patient selection (Figure 3).

### 5.5 Mechanisms of Acquired Resistance and Future Directions

PKMYT1 upregulation represents a key mechanism of resistance to MK‐1775 (WEE1 inhibitor) [72]. Potential resistance mechanisms to PKMYT1 inhibitors include bypass pathway activation, drug efflux, or compensatory upregulation of other CDK1 regulators (e.g., CDC25, WEE1). Strategies to overcome resistance may include intermittent dosing, triple combinations (e.g., PKMYT1/ATR/PARP inhibition), or PROTAC‐mediated degradation. Future work should explore whether MMB‐FOXM1‐driven transcription or other replication stress signatures can expand the range of tumors benefiting from PKMYT1 inhibition [63, 68].

**FIGURE 4:**
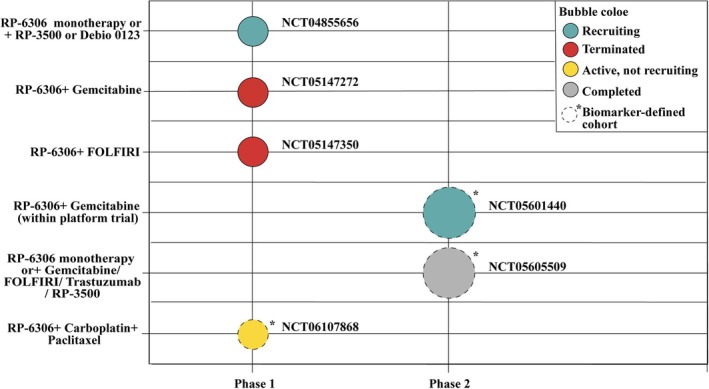
Clinical development of PKMYT1 inhibitor lunresertib (RP‐6306). This landscape summarizes six registered RP‐6306 trials by phase, regimen, and status. Bubbles are color‐coded by trial status (green = recruiting; red = terminated; yellow = active; gray = completed). Size indicates enrollment; dashed borders denote biomarker‐defined cohorts. Phase 1: NCT04855656 evaluates RP‐6306 ± ATR inhibitor (RP‐3500) or SHP2 inhibitor (Debio 0123) in advanced solid tumors (recruiting). Two chemotherapy combinations (gemcitabine, FOLFIRI) were terminated, suggesting toxicity or strategic reprioritization. NCT06107868 targets TP53‐mutant ovarian/uterine cancers (active). Phase 2: NCT05605509 (completed) assessed RP‐6306 combinations with biomarker‐response correlation. NCT05601440 is a recruiting platform trial for CDK4/6 inhibitor‐resistant ER+/HER2‐breast cancer using liquid biopsy‐guided stratification.

In summary, PKMYT1 inhibition represents a promising precision medicine strategy, with RP‐6306 leading clinical development and multiple novel agents in preclinical evaluation. Rational biomarker‐driven patient selection and combination strategies will be critical to maximizing therapeutic benefit (Table 2, Figure 4).

**TABLE 2:** Clinical trials of PKMYT1 inhibitors (focus on RP‐6306).

NCT number	Study title	Phase	Combination regimen	Biomarker‐defined cohort	Indication(s)	Status	Results available
NCT04855656	Study of Lunresertib alone or in combination with RP‐3500 or Debio 0123 in patients with advanced solid tumors	Phase 1	RP‐6306 monotherapy or + RP‐3500 or Debio 0123	No	Advanced solid tumors	Recruiting	No
NCT05147272	study of RP‐6306 with gemcitabine in advanced solid tumors	Phase 1	RP‐6306 + Gemcitabine	No	Advanced solid tumors	Terminated	Yes
NCT05147350	Study of RP‐6306 with FOLFIRI in advanced solid tumors	Phase 1	RP‐6306 + FOLFIRI	No	Advanced solid tumors	Terminated	Yes
NCT05601440	Liquid‐biopsy informed platform trial to evaluate CDK4/6‐inhibitor resistant ER+/HER2‐ metastatic breast cancer	Phase 2	RP‐6306 + Gemcitabine (within platform trial)	Yes (CDK4/6i‐resistant ER+/HER2‐mBC)	Metastatic breast cancer	Recruiting	No
NCT05605509	RP‐6306 in patients with advanced cancer	Phase 2	RP‐6306 monotherapy or + Gemcitabine/FOLFIRI/Trastuzumab/RP‐3500	Yes (exploratory biomarkers)	Advanced cancer	Completed	No
NCT06107868	Phase 1 study of RP‐6306 with carboplatin and paclitaxel in TP53 ovarian and uterine cancer	Phase 1	RP‐6306 + Carboplatin + Paclitaxel	Yes (TP53 mutation)	Ovarian/uterine cancer	Active, not recruiting	No

## 6 Future Perspectives and Unresolved Controversies

Despite significant progress in elucidating PKMYT1 biology, several critical challenges remain. First, the context‐dependent role of PKMYT1 requires clarification. While predominantly oncogenic, PKMYT1 functions as a tumor suppressor in a subset of LUAD by inhibiting AKT1 phosphorylation [43]. Understanding the molecular determinants‐such as specific mutation backgrounds or post‐translational modifications‐that dictate this dichotomy is essential for accurate patient stratification. Second, mechanisms of acquired resistance to PKMYT1 inhibitors remain largely unexplored. Unlike WEE1 inhibition, where PKMYT1 upregulation mediates resistance [72], the bypass pathways or compensatory mechanisms emerging under PKMYT1‐targeted pressure are unknown. Third, biomarker development must evolve beyond CCNE1 amplification. Composite signatures incorporating TP53 status, ER expression, PKMYT1/WEE1 ratios, and replication stress markers may better identify responsive populations. Finally, optimizing combination strategies‐particularly with ATR inhibitors, immunotherapy, or PARP inhibitors‐requires careful management of overlapping toxicities. Emerging technologies, such as PROTAC‐mediated degradation [68], may overcome limitations of catalytic inhibition and provide new avenues for treating resistant malignancies. Addressing these questions will be pivotal for translating PKMYT1 targeting into a precision oncology paradigm.

## Author Contributions

**Rongzhang He:** funding acquisition, methodology, resources, writing – review and editing. **Shunxin Song:** funding acquisition, methodology, resources. **Lingxi Li:** conceptualization, investigation, methodology, writing – original draft. **Binfan He:** investigation, methodology. **Mengmeng Hao:** project administration, visualization. **Ye Liu:** conceptualization, data curation.

## Funding

This work was supported by the Health Research Project of Hunan Provincial Health Commission (W20242017, 20255911).

## Ethics Statement

The authors have nothing to report.

## Consent

The authors have nothing to report.

## Conflicts of Interest

The authors declare no conflicts of interest.

## Data Availability

Data sharing not applicable to this article as no datasets were generated or analysed during the current study.
